# Japanese mobile phone study

**DOI:** 10.1038/sj.bjc.6604397

**Published:** 2008-05-27

**Authors:** B Hocking

**Affiliations:** 1 Specialist in Occupational and Environmental Medicine, 9 Tyrone Street, Camberwell, Victoria 3124, Australia

**Sir,**

Takebayashi *et al* (2008) have conducted a case–control study of 322 cases of brain tumour (glioma, meningioma and pituitary adenoma) and found no association with mobile phone usage. There are several flaws in the methodology of their study, which would lead to a null finding and the risk of a type-2 (false negative) error.

Cases were recruited from hospitals in the Tokyo area, which, it was estimated, treat 75% of all brain tumour cases. The participation rate for the glioma cases was 59%, which was only 43% of the total cases in Tokyo, and slightly higher for the other tumours. It is remarkable that none of the glioma cases were reported as having died or were so incapacitated that a proxy (eg, a spouse) was used for reporting phone usage. This is important because data from proxies are unreliable.

Exposure data are calculated from interviews using the Interphone protocol. Information on lifetime history of use of mobile phone was sought from subjects, including the average duration and frequency of calls, the make and model of phones used and the side of the head in contact with the phone. From these data, cumulative length of use and cumulative call time were calculated. The authors then combined these data with the calculated SAR of the various phones to give further measures of exposure. However, the accuracy of the key data regarding remembrance of phone usage and call times over past years was not confirmed from billing data or other sources. The Interphone study, along with other studies, has found that recall of phone use even over previous months is inaccurate and may be associated with random errors, leading to overestimation or under-estimations of true usage (Parslow *et al*, 2003; Samkange-Zeeb *et al*, 2004; Shum *et al*, 2005; Vrijheid *et al*, 2006). It is of concern that the authors do not refer to Interphone's own validation study, which highlights this crucial methodological problem. Using such inaccurate data for estimating cumulative exposures or for combining with SAR data, will lead to a null finding.

The controls were selected from the community. They contain proportionately more subjects who had college education than the cases (Table 1); education was used as a proxy for socioeconomic status. The better educated and, therefore, control subjects with higher socioeconomic status are likely to have been bigger users of mobile phones because they can better afford them and may be provided with phones free of cost in the course of their higher status work. This increased exposure will obscure any effect in the cases.

It was found that the odds ratio for glioma patients in the most heavily exposed group was 5.84 (0.96–35.60) (Table 3). The authors dismiss this finding as recall bias due to persons with a tumour seeking to attribute it to mobile phone use. However, the odds ratio for the most exposed meningioma cases was only 1.14 (0.28–4.6). If recall bias is the true explanation for the increased risk of glioma, it should similarly have affected the meningioma group, but it has not. Therefore, the increased risk in the glioma group may be a true finding.
